# Supplementary material for: SOX2 and NR2F1 coordinate the gene expression program of the early postnatal visual thalamus
Source: Biol Open. 2025 Aug 1;14(8):bio062014. doi: 10.1242/bio.062014 (PMC12352279; doi:10.1242/bio.062014)
Supplement: Supplementary information [file biolopen-14-062014-s1.pdf]

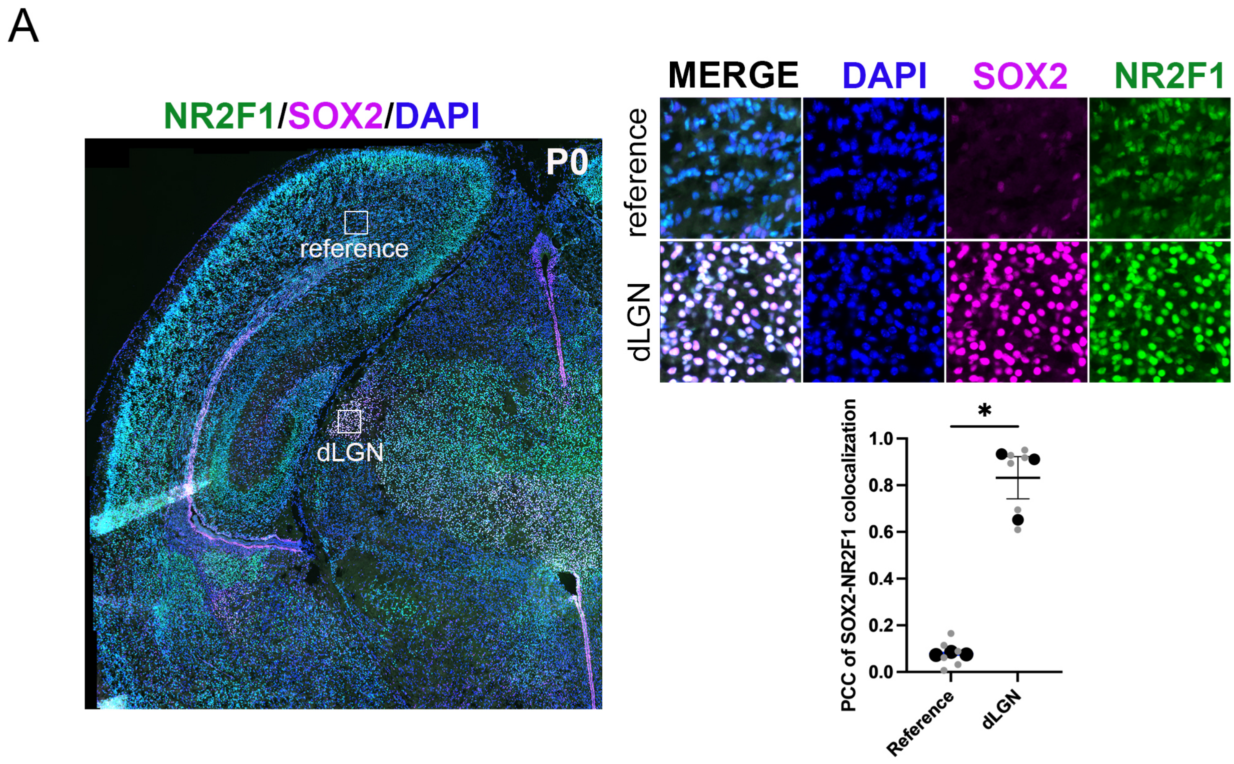

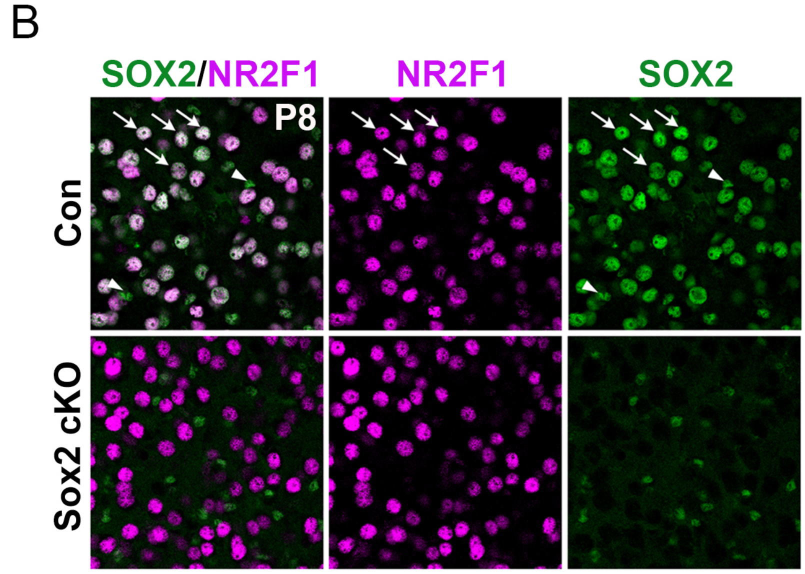

Fig. S1. SOX2 and NR2F1 are co-localized in the dLGN at P0 and P8

A. IF with antibodies recognizing SOX2 (magenta) and NR2F1 (green) at P0 on coronal sections. A detail of this IF is shown in **Fig.1A.** Squares indicate the region of interest analyzed in the dLGN and in a reference area for the quantification of co-localization of SOX2 and NR2F1 shown in the graph. Large dots represent the mean intensity for each brain analyzed and the small dots represent each individual measurement. Mean +/- s.e.m. is indicated. $*P < 0.05$, $**P < 0.01$, $***P < 0.001$; unpaired t-test.

B. dLGN IF with antibodies recognizing SOX2 (green) and NR2F1 (magenta) at P8 on coronal section of controls (*Sox2$^{flox/flox}$* or *Sox2$^{flox/+}$*), and thalamic *Sox2* mutant (cKO). Note that *Sox2* thalamic loss (*Sox2* cKO in the P8 panel) does not detectably affect NR2F1 expression. Scale bar: 50μm.

Biology Open • Supplementary information

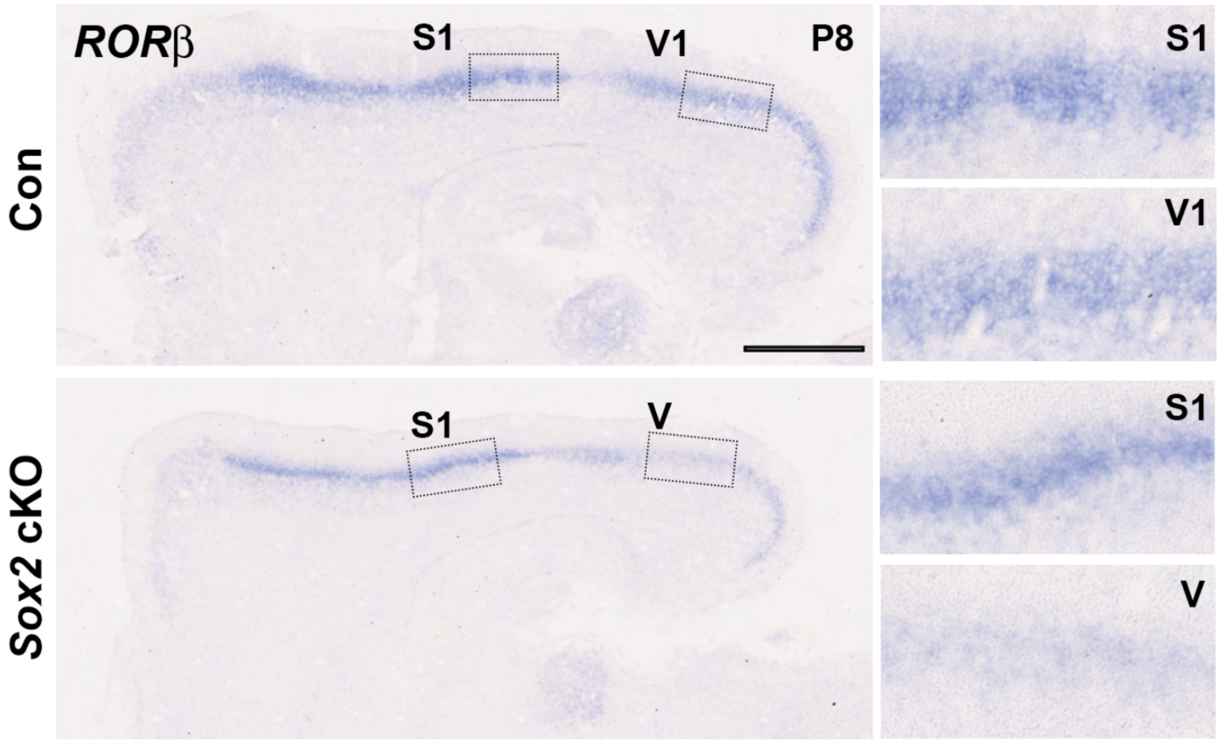

Fig. S2. ***Sox2*** **thalamic cKO affects the development of cortical layer 4th**
ISH with a *Rorβ* probe, marking layer 4, on sagittal brain sectionsprobe
halamic *Sox2* mutants (*Sox2* cKO) versus controls carrying wild type *Sox2* (Con: $Sox2^{flox/flox}$ or $Sox2^{flox/+}$) at P8 shows a reduction in the region corresponding to the visual (V,V1) and somatosensory (S1) areas (enlarged details). The results shown are representative of n=3 mutant and n=3 control brains analysed. Scale bar: 1mm.

Biology Open • Supplementary information

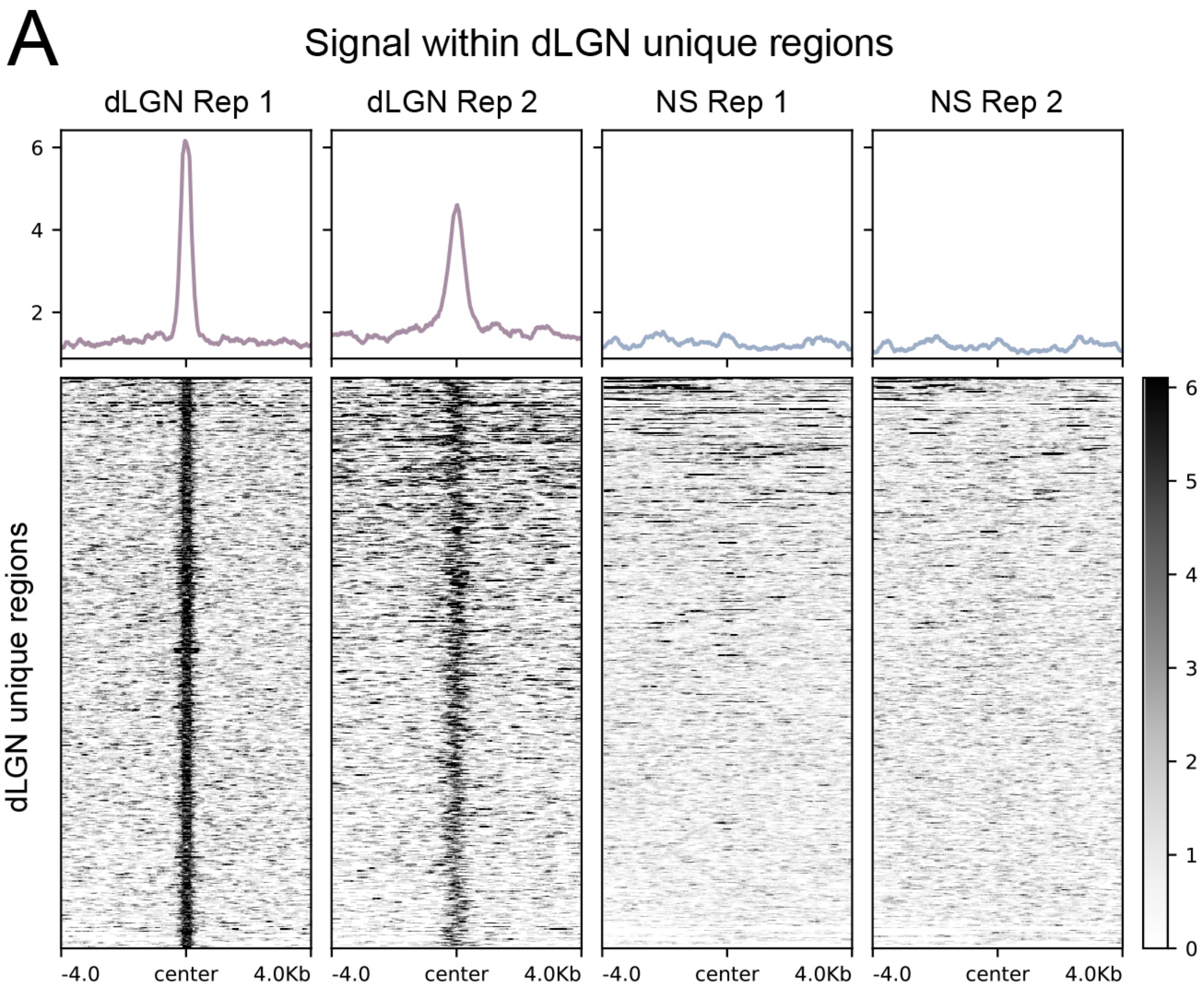

Fig. S3. dLGN-specific SOX2 peaks

A. Average signal profiles and intensity heatmaps showing SOX2 CUT&RUN signal from the dLGN and Neurospheres within the dLGN unique peak regions. dLGN shows reproducible signal, whereas neurosphere datasets are devoid of enrichment over background. Signal normalized with RPGC. NS = Neurospheres.

Biology Open • Supplementary information

Table S1.

Available for download at
https://journals.biologists.com/bio/article-lookup/doi/10.1242/bio.062014#supplementary-data

Table S2.

Available for download at
https://journals.biologists.com/bio/article-lookup/doi/10.1242/bio.062014#supplementary-data

Table S3.

Available for download at
https://journals.biologists.com/bio/article-lookup/doi/10.1242/bio.062014#supplementary-data

Table S4.

Available for download at
https://journals.biologists.com/bio/article-lookup/doi/10.1242/bio.062014#supplementary-data
